# *Bacillus amyloliquefaciens* AK-12 Helps Rapeseed Establish a Protection against *Brevicoryne brassicae*

**DOI:** 10.3390/ijms242115893

**Published:** 2023-11-02

**Authors:** Shixiong Qian, Ayesha Ahmed, Pengbo He, Pengfei He, Shahzad Munir, Mengyuan Xia, Chaoyun Tang, Ping Tang, Zaiqiang Wang, Rizwan Khan, Xingyu Li, Yixin Wu, Yueqiu He

**Affiliations:** 1 State Key Laboratory for Conservation and Utilization of Bio-Resources in Yunnan, Yunnan Agricultural University, Kunming 650201, China; 2 Faculty of Agronomy and Biotechnology, Yunnan Agricultural University, Kunming 650201, China

**Keywords:** rapeseed, *Brevicoryne brassicae*, *Bacillus*, biopesticide, induced resistance

## Abstract

Aphids are a serious threat to rapeseed (*Brassica napus* L.) production, and cause unmanageable loss. Therefore, effective prevention and management strategies are urgently required to avoid losses. *Bacillus amyloliquefaciens* AK-12 isolated from a dead aphid with aphicidal activity was tagged with a green fluorescent protein through a natural transformation. The transformed strains were checked for stability and growth, and the best-performing strain was tested for its colonization inside and outside the rapeseed plant. The stability of AK-12-GFP reached more than 95%, and the growth curve was consistent with that of AK-12. After 30 days of treatment, the colonization of 1 × 10^6^ CFU/g was recorded in rapeseed leaves. Interestingly, AK-12 reduced the aphid transmission rate compared with the control and improved the growth of the rapeseed seedlings. Meanwhile, the AK-12 strain also exhibited phosphorus, potassium-solubilizing, and nitrogen-fixing activity, and produced 2.61 µg/mL of IAA at 24 h. Regulation in the activity of four enzymes was detected after the AK-12 treatment. Phenylalanine ammonia lyase (PAL) was recorded at a maximum of 86.84 U/g after 36 h, and catalase (CAT) decreased after 48 h; however, peroxidase (POD) and polyphenol oxidase (PPO) reached the maximum within 12 h of AK-12 application. Additionally, important resistance genes related to these enzymes were upregulated, indicating the activation of a defense response in the rapeseed against aphids. In conclusion, defense enzymes and defense-related gene activation could improve the pest resistance in rapeseed, which has good application prospects for the future to be developed into biopesticide.

## 1. Introduction

Rapeseed (*Brassica napus* L.) is the third largest oil crop and holds enormous economic and nutritional value around the globe [1]. At present, the area under the cultivation of rapeseed is 17 million hectares, and the total output is more than 24 million tons per annum worldwide. The main producing countries are China, Canada, India, and others [2]. The crop is an important source of edible vegetable oil and protein [3]. Cabbage aphids (*Brevicoryne brassicae* (Linnaeus) are considered one of the most important pests that cause serious damage to the leaves, flowers, and kernels of rapeseed [4].

At present, the main measures to control aphids include agronomic practices [5,6,7,8] and chemical [9,10,11], physical [12,13,14], and biological control. The application of chemical insecticides has been the most-used strategy till now. The use of chemical pesticides has many drawbacks, such as environmental pollution, the development of resistance in pests, and harm to human health [15]. Therefore, it is imperative to devise environmentally friendly solutions and microbial agents with insecticidal activity may serve as better control options. Entomopathogenic fungi, such as *Fusarium semitectum*, *Metarhizium anisopliae*, and *Lecanicillium lecanii*, have been shown to be efficient against aphid prevention and control due to their culturable characteristics and wide insecticidal spectrum [16,17,18]. In addition, *Bacillus atrophaeus* [19], *B. amyloliquefaciens* [20], and *Pseudomonas* sp. [21,22] have also been reported to exhibit aphicidal activity. Three different *B. amyloliquefaciens* strain cell suspensions were used against the peach–potato aphid (*Myzus persicae*), with 100% mortality [20]. The current opportunities and demands for effective biological control are greater than ever before, considering the fact that biocontrol programs are generally less frequently performed in developing countries [23,24].

*Bacillus* is considered one of the most extensively studied plant-beneficial bacteria for its disease control and growth-promoting efficiency. *Bacilli* are prioritized due to their efficient colonization and defense activation in host plants [15,25]. Plant growth promotion using beneficial bacteria is mainly controlled through the secretion of plant hormones and by increasing the availability and uptake of nutrients. These bacteria are known to produce auxins such as indole-3-acetic acid, which stimulate the growth of lateral roots and root hairs, thereby increasing the surface area for nutrient uptake [26]. Several *Bacilli* are known to fix nitrogen and solubilize phosphate and potassium. The enhanced availability and assimilation of nutrients are directly related to growth promotion and improved vigor, which indirectly protect plants from deleterious effect of pathogens and insect pests [27]. In addition to plant growth promotion, *Bacilli* can boost plant defense through the induction of resistance, which enables the plant to mount a swift defense response against pests/pathogens [28]. For instance, *Bacillus megaterium* induced resistance in a tea plant by upregulating defense-related enzymes, such as -peroxidase, chitinase, β-1,3-glucanase, and phenyl alanine ammonia lyase [29]. Therefore, it was hypothesized that the application of a biocontrol agent may reduce the aphid infestation by activating the plant defense response and improving plant vigor.

In this regard, the present study aimed at using an efficient biocontrol agent isolated from dead aphids to control the *B. brassicae* aphid and to explore the colonization dynamics of *Bacillus* strains in rapeseed, as well as the defense enzymes and genes involved in the resistance of plants against aphids. To our knowledge, there have been no reports of using the colonization dynamics of microbial resources to repel and control aphids, as well as to improve the crop resistance against aphids.

## 2. Results

### 2.1. Comparison of Strain Stability and Growth Curves

**Figure 1:**
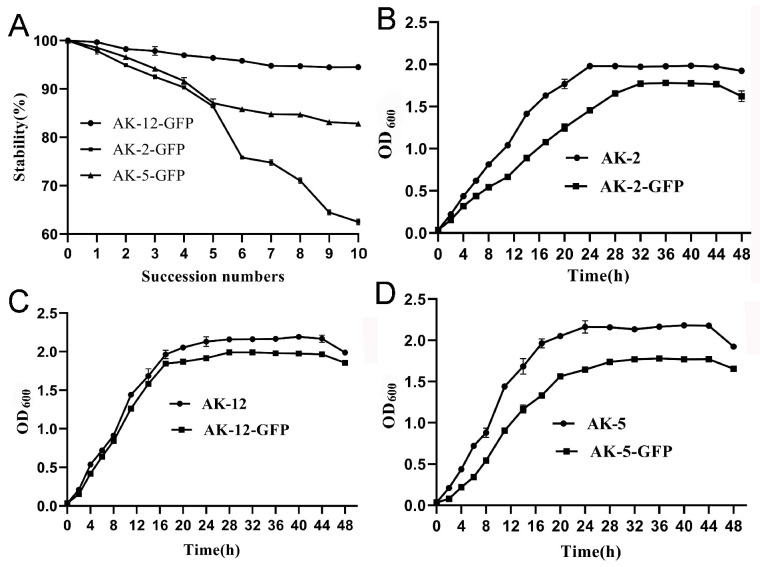
Stability and growth curves of the bacterial strains. (**A**) Stability of different GFP-tagged strains. (**B**) Growth curves of AK-2 wild type and GFP, (**C**) AK-12 and GFP, (**D**) and AK-5 and GFP.

The natural transformation was used to successfully introduce green fluorescent protein into *Bacillus* AK-2, AK-5, and AK-12 competent cells, and the transformants grew in the LB agar medium supplemented with 10 μg/mL chloramphenicol. All the three strains showed nearly the same fluorescence under a UV chamber. The results showed that with the increase in transduction times, the number of strains that lost fluorescence began to increase, but AK-12-GFP showed a very stable fluorescence, reaching more than 95.00% after 10 generations. AK-5 also showed a rapid fluorescence decline in the first five generations, but the decline was slower afterwards, and achieved an 84% stability till the tenth generation. However, AK-2 was not as stable as the former two, with only 62% at the tenth generation, showing a rapid loss that could further explain the presence of negative transformants in AK-2 during the transformation (Figure 1). Furthermore, the growth rate of the labeled strains was lower than that of the wild-type strains, and the number of bacteria was not the same as that of the wild-type. This may have been due to the introduction of the green fluorescent protein vector that increased the metabolic burden of the strain. This negative effect was less pronounced in AK-12, as compared to AK-5 and AK-2. Due to the higher stability, AK-12 was used for further experiments.

### 2.2. Colonization Dynamics of AK-12

**Figure 2:**
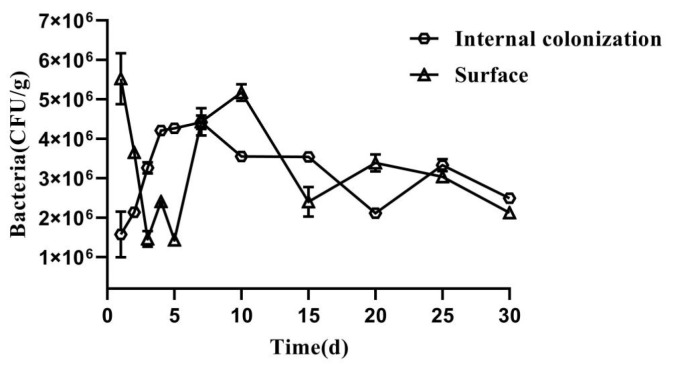
External and internal colonization capacity of the AK-12 strain in rapeseed.

In order to study the colonization efficiency of AK-12, the number of fluorescent bacteria on and inside the leaves was counted at 1–5, 7, 10, 15, 20, 25, and 30 days after treatment (Figure 2). The colonization of AK-12 gradually increased from 1 to 7 days before reaching the highest value and, then, fluctuated at 3 × 10^6^ CFU/g with a significant downward trend after 25 days. The number of bacteria on the leaf surface reached the maximum after the first day, followed by a sharp decline.

### 2.3. Strain Colonization Ability to Establish Green Barrier and Control Cabbage Aphids

#### 2.3.1. Effect of Strains on Cabbage Aphid Transmission

**Figure 3:**
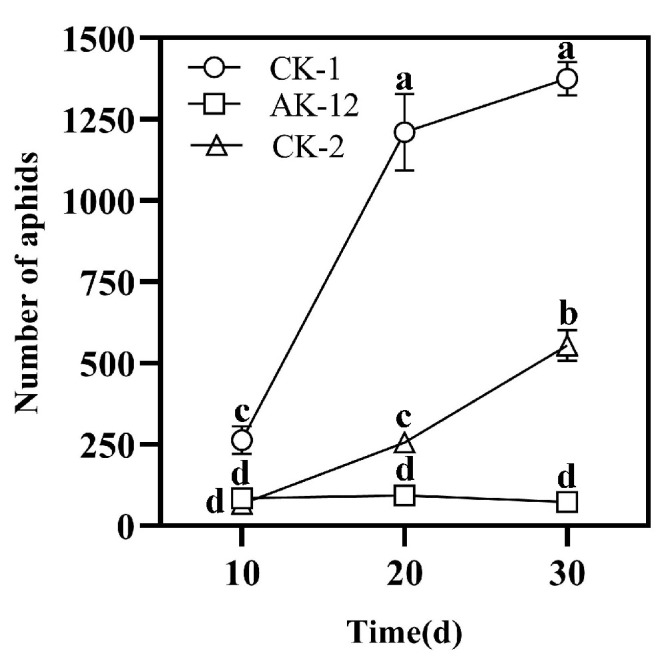
Aphid transmission across AK-12-treated and control pots. Note: different letters indicate statistical differences (*p* < 0.05).

**Figure 4:**
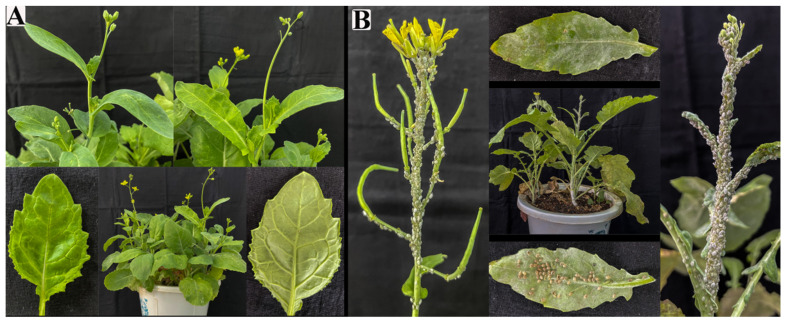
Comparison of AK-12 (**A**) treatment and CK-1 (**B**) rapeseed growth.

The colonization of AK-12 stabilized after 5 days of treatment; therefore, 100 aphids were seeded in the third row after 5 days and the number of aphids in the treated and control plants was recorded every 10 days. The results showed that the number of aphids in the treated plants was lower than in the control plants. There was no significant difference between the number of aphids in the AK-12-treated and CK-2 plants at 10 days, while after 20 days, the aphids in the control plants were significantly higher than that in the treated plants, showing rapid growth and transmission. The results showed that AK-12 could significantly reduce the aphid transmission rate. The number of aphids in CK-2 was lower than that of CK-1, and the growth rate was also significantly lower than that of CK-1 (Figure 3). Few aphids were observed only on the leaves of plants treated with AK-12 (Figure 4A), whereas control plants were highly infested with aphids and severely damaged (Figure 4B). At the same time, a severe powdery mildew infection was observed in the control plants (Figure 4B), while the treated plants did not exhibit any disease symptoms. In the previous experiments, we tested the fungal inhibition activity of AK-12, and no antifungal activity was detected. Therefore, we speculated that the AK-12 strain may have induced resistance in the rapeseed plants, which protected the plant from a fungal attack.

#### 2.3.2. Effect of AK-12 on Cabbage Aphid Feeding

**Figure 5:**
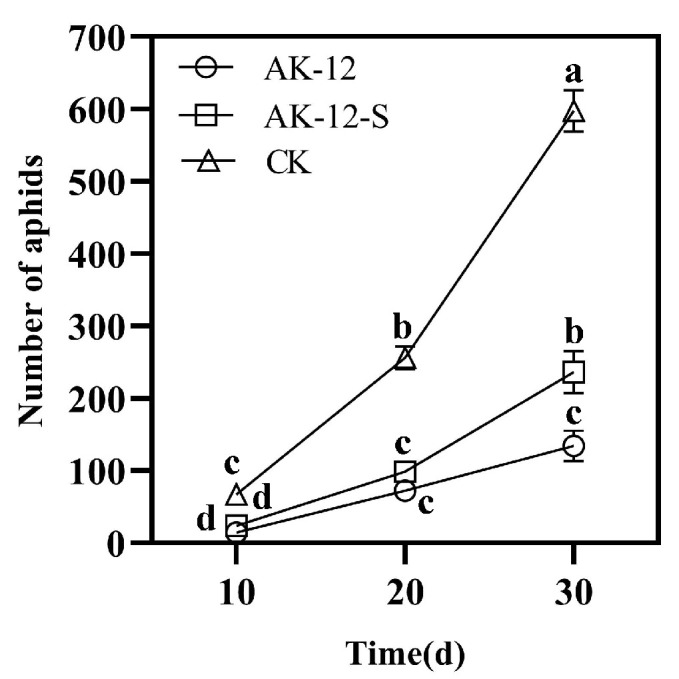
Degree of aphid infestation in different treatments. Note: different letters indicate statistical differences (*p* < 0.05).

**Figure 6:**
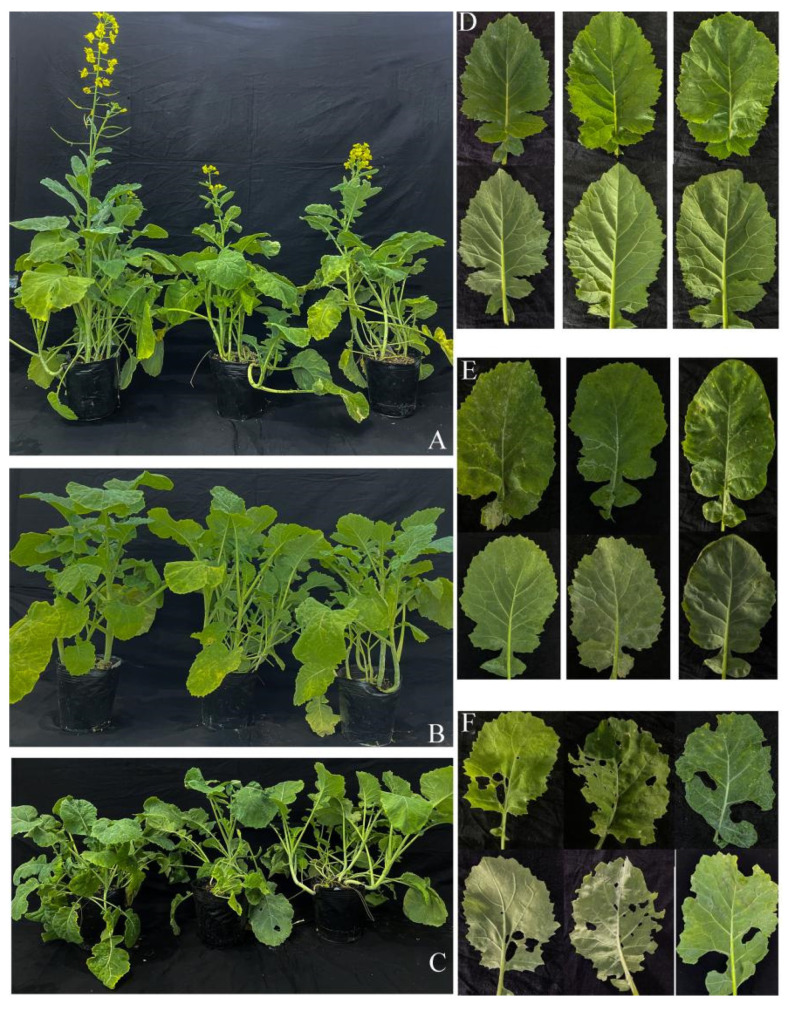
Comparison of rapeseed growth and leaf health under different treatments, where (**A**,**D**), (**B**,**E**) and (**C**,**F**) represent AK-12, AK-12-S, and control, respectively.

For both the AK-12 bacterial suspension or supernatant treatment, the number and growth rate of aphids at different time points were lower than the control group, with up to 630 aphids/basin (Figure 5), while the plants treated with the suspension (Figure 6A) and supernatant (Figure 6B) showed better growth after 30 days, as compared with the control plants. The control rapeseed plants (Figure 6C) grew slowly and collapsed, and the leaves were scattered; other pests besides the large number of aphids were also observed.

### 2.4. Growth Promoting Effect of AK-12 on Rapeseed Plants

**Figure 7:**
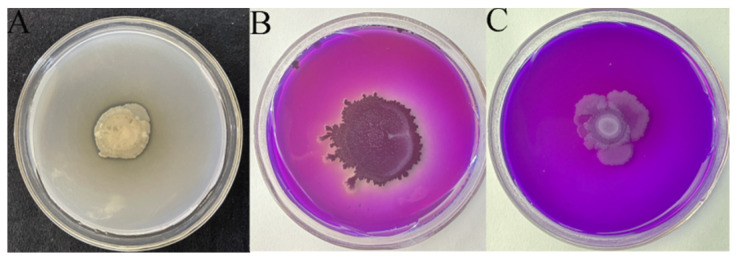
AK-12-solubilized phosphorus (**B**), potassium solution (**C**), and fixed nitrogen (**A**).

**Figure 8:**
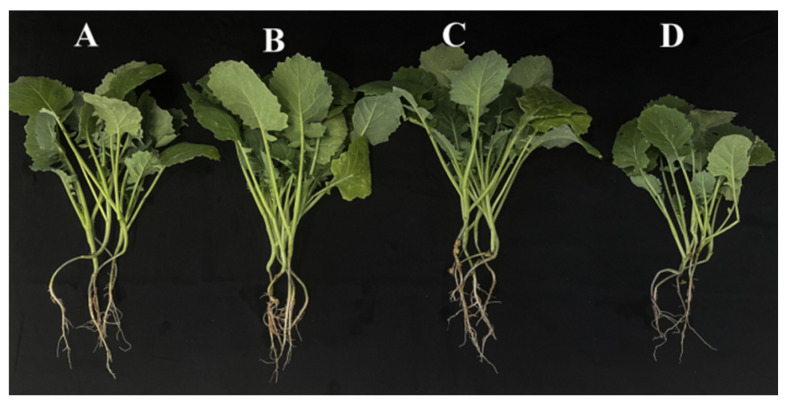
Growth-promoting effect of AK-12 on rapeseed seedlings; AK-12 was applied through (**A**) foliar spray, (**B**) root irrigation, and (**C**) foliar spray and root irrigation, and (**D**) water was used as a control.

The attributes related to the growth-promoting characteristics for AK-12 were tested, and was shown that it could solubilize P and K and fix N (Figure 7). Further, IAA production of AK-12 in a 24 h grown culture was quantified to be 2.61 µg/mL, which also highlighted its potential in plant growth promotion. The plant height, stem width, fresh weight, and dry weight of each rapeseed plant were measured. The growth status (Figure 7) and physiological indicators were higher in plants treated with AK-12 than the control; however, there was no significant difference between the plants treated with foliar spray, root irrigation, or both (Figure 8, Table 1).

### 2.5. Induced Resistance Studies of Strain

**Figure 9:**
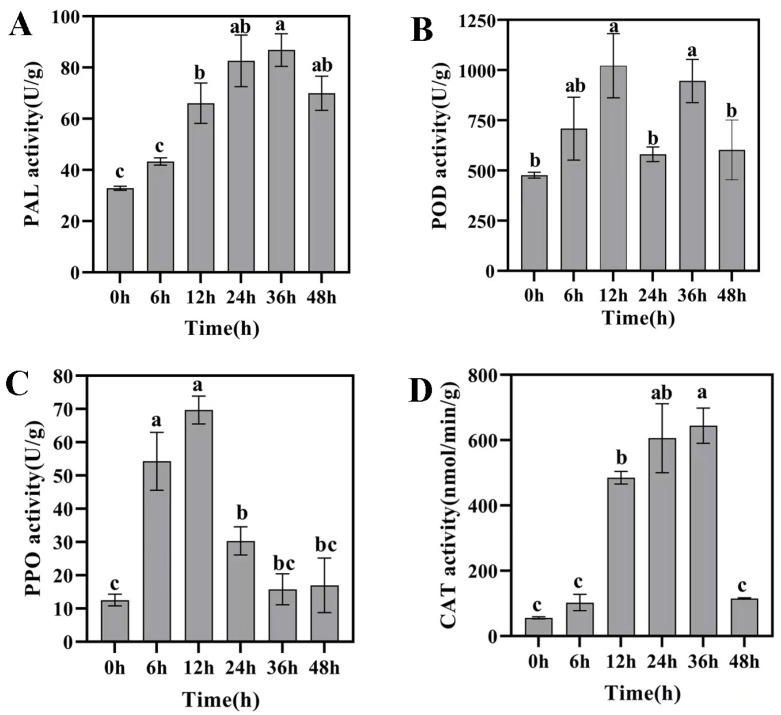
Changes in defense enzyme activity in rapeseed. (**A**) PAL enzyme activity. (**B**) POD enzyme activity. (**C**) PPO enzyme activity. (**D**) CAT enzyme activity. Note: different letters indicate statistical differences (*p* < 0.05).

Peroxidase (POD), phenylalanine ammonia lyase (PAL), polyphenol oxidase (PPO), and other defense enzymes play a great role in resistance. After the treatment of the AK-12 strain, the activity of the four enzymes greatly improved (Figure 9). Among them, the PAL activity increased after the treatment, reached the maximum of 86.84 U/g at 36 h, and started to decline at 48 h. The catalase activity (CAT) also followed the same trend, except that the activity of the CAT enzyme decreased sharply at 48 h and was not significantly different from 0 h. Peroxidase (POD) and polyphenol oxidase (PPO) activity both reached the maximum after 12 h of treatment, but POD fluctuated after this time, while PPO decreased and, then, approached the same level as 0 h. Therefore, the AK-12 strain significantly improved the defense enzyme activity and improved the pest resistance in rapeseed.

**Figure 10:**
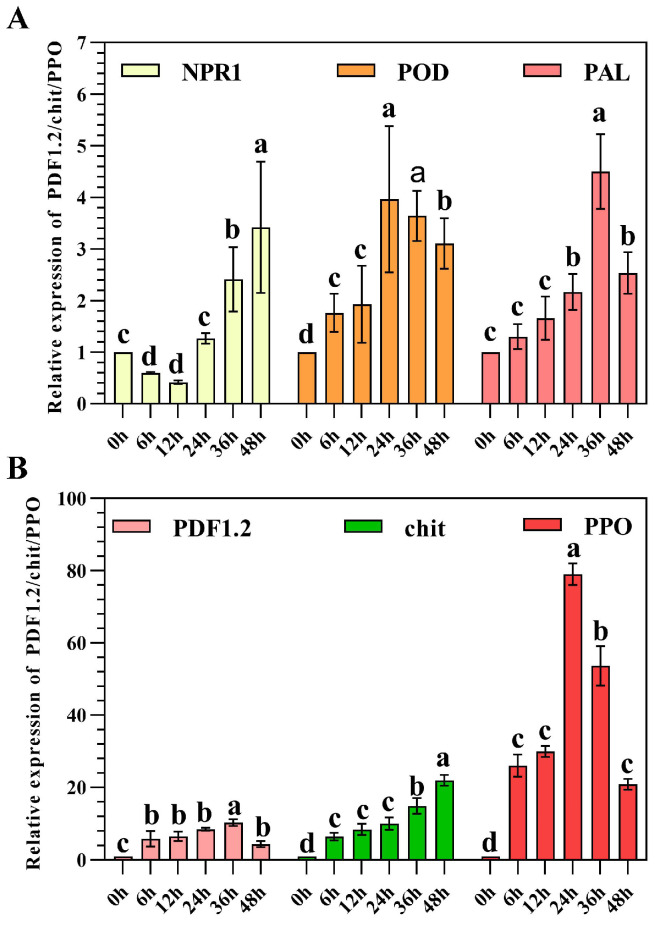
The expression of resistance-related genes induced using the AK-12 strain. (**A**) Relative expression of NPR1, POD, and PAL. (**B**) Relative expression of PDF1.2, chit, and PPO. Note: different letters indicate statistical differences (*p* < 0.05).

After treatment with AK-12, the expression of NPR1 at 6–12 h was downregulated, which was significantly lower than that of 0 h of the control treatment, whereas the expression at 36–48 h was significantly higher than that of 0 h. The PDF gene was upregulated within 72 h, which was significantly higher than 0 h. The expression was stable, reaching the maximum at 36 h, and the expression was 11 times that of the 0 h control. The chitinase gene was 6–30 fold upregulated after the AK-12 treatment, with the maximum expression at 48 h, as compared with 0 h of the control treatment. While the POD and PAL genes were upregulated throughout the trial and showed a trend of rising first and then decreasing, the difference was that the POD gene reached the maximum at 24 h, while the PAL gene was highly expressed at 36 h (Figure 10). Moreover, the expression of the PPO gene was 20–82 times higher at 6 h to 48 h than the 0 h of the control treatment, and the AK-12 treatment showed the highest effect on the PPO gene expression. After the AK-12 treatment, POD, PAL, PPO, and the other resistance-related genes were also upregulated, which further explained the intrinsic mechanism of the AK-12-induced resistance in rapeseed.

## 3. Discussion

Aphids are one of the major threats to sustainable agricultural production. In order to protect crops from damage, farmers tend to apply large quantities of pesticides, which are neither cost-effective nor beneficial for plant and environmental health. In this regard, plant-associated beneficial bacteria have gained much attention for their biocontrol properties. Previous studies have also shown the great potential of *Bacillus* sp. for aphid biocontrol [30].

The application of plant growth-promoting rhizobacteria (PGPR) is an environmentally friendly alternative to the chemicals used in agricultural production and crop protection. The mechanisms by which PGPR colonizes the rhizosphere include the recognition of chemical signals and nutrients in root exudates, antioxidant activity, biofilm production, bacterial motility, and the efficient evasion and suppression of the plant immune system. One of the approaches that can be used to solve some of the current agricultural problems is the application of beneficial microorganisms naturally associated with plants, which do not pose deleterious effects on the environment, human health, and animals [31,32,33]. In the present study, *Bacillus* strains were tagged with GFP and the most stable strain was further employed to study the antagonistic action against aphids. Among all, AK-12-GFP showed stable growth in culture media; it could also colonize both on the leaf surface and internal tissue as an endophyte. However, the endophytic colonization was more stable, as it was at the maximum until 3 weeks and slowly declined over the course of time. The stability of the internal colonization is attributed to the protective environment inside the plant tissue, which might have shielded the bacteria from fluctuations of the external environment. *Bacillus* strains are efficient colonizers of plants; for instance, the citrus endophytic strain *Bacillus subtilis* L1-21 can colonize in citrus, green soybean, tomato, green bristle grass, grapes, chili, Sonchusoleraceus, Malvastrum, eggplant, and *Malva verticillata*, indicating colonization in multiple plant hosts [34].

Moreover, microbial competition and successful colonization in crops should be considered in the development and application of biocontrol agents [35]. The relatively stable colonization of the introduced biocontrol agent in plant is considered important for improving plant growth and resistance. Our results showed that the number of aphids increased after 20 days due to a decline in AK-12 colonization. Therefore, it is necessary to ensure that the colonization of AK-12 remains stable at 3 × 10^6^ CFU/g for the efficient inhibition of aphid growth. Furthermore, the application of AK-12 also hindered the aphid transmission to other plants (Figure 3), as a higher number of aphids was recorded in CK-1 plants, which were adjacent to the plants infested with aphids, whereas the number of aphids was lower in CK-2 plants, which were adjacent to the AK-12-treated plants. Interestingly, the application of AK-12 not only reduced the transmission, but also compromised the feeding ability of the aphids. The damage in the treated plants was minimal, whereas the untreated plants were severely damaged due to aphids feeding on plant tissues (Figure 6).

PGPR are also well-known for their growth-promoting effects on plants, which they achieve by producing plant growth-promoting hormones and helping plant in nutrient assimilation [36]. AK-12 was found to promote growth in rapeseed plants and its IAA production was quantified to be 2.61 µg/mL, as IAA improves the root hair growth and lateral root development, which increases the surface area for nutrient uptake. For instance, the inoculation of *B. licheniformis* PR2 enhanced the growth parameters of poplar seedlings and the strain could also produce IAA [37]. A recent study also reported the involvement of IAA in the growth promotion of maize using *B. thuringiensis*, as the knock-out mutant of the IAA gene lost the ability to promote growth [38]. The plants treated with AK-12 through foliar application and root irrigation exhibited higher growth indexes as compared with the control plants. The growth promotion could also be attributed to the improved nutrient availability, as AK-12 could solubilize phosphorus and potassium, as well as fix nitrogen. The enhanced nutrient assimilation increased the photosynthetic rate, thereby improving the plant growth. For example, increased nitrogen absorption improves the chlorophyl content, which promotes photosynthesis and biomass production of the plant [26]. Furthermore, the increase in phosphorus availability though the solubilization of inorganic phosphate is also known to increase root density and nutrient uptake by plants [39]. After nitrogen and phosphorus, potassium is the most important plant nutrient that plays a key role in the growth, metabolism, and development of plants. Potassium-solubilizing bacteria are considered significantly important for crop growth [40].

PGPR can be beneficial for plants in various ways, not merely limited to growth promotion. The application of biocontrol bacteria is known to systematically activate the plant’s immune system. In the absence of pathogen, primed plants do not show major changes in the expression of defense-related genes, but generate swift defense responses against pathogen or insect attacks, providing broad-spectrum resistance [41,42]. The application of biocontrol agent prior to aphid infestations can provide better prevention through the induction of plant resistance against pests and pathogens. In this regard, some important enzymes, such as PAL [42,43,44], CAT [45], PPO [46], and POD [47], have been shown to be involved in plant resistance against pests and diseases. However, these enzymes are also activated upon pest infestation as the plant’s defense mechanism, but primed plants also exhibit similar responses. Therefore, in order to confirm the induction of resistance, AK-12 was applied to the rapeseed plants prior aphid infestation. The defense enzymes and genes involved in the resistance displayed maximum values that greatly improved the resistance inside the host plants against invading enemies. The time of applying the bioagent is very important for mounting a swift defense response against plant pests.

In conclusion, we suggest that AK-12 is a potential biocontrol strain with diverse interactions inside host plants in the presence of aphids. AK-12 did not only provide growth-promoting effects, but also induced defense against pests. Furthermore, after the AK-12 treatment, early flowering in rapeseed was observed, which requires a further in-depth study.

## 4. Materials and Methods

### 4.1. Materials, Bacterial Strains, and Reagents

Sunshine 3 rapeseed, widely cultivated in central Yunnan, was grown in pots. *Bacillus subtilis* AK-2, AK-5, and *B. amyloliquefaciens* AK-12 were isolated from naturally dead aphids. After greenhouse and field experiments, it was determined that it had aphicidal activity against cabbage aphids (*Brevicoryne brassicae*) and *Pemphigus betae*. The plasmid pHT01-P43GFPmut3a carrying the GFP protein gene was maintained in the Molecular Plant Pathology Lab at Yunnan Agricultural University, Kunming, China.

Cabbage aphids were collected from an organic vegetable base in Yunnan Yunling Fresh living Co., Ltd., Kunming, China. *B. napus* was inoculated in the National Engineering Research Center of Agricultural Biodiversity Application Technology of Yunnan Agricultural University. More than 1000 wingless adult aphids were selected and inoculated on potted rapeseed seedlings. Rapeseed seedlings were replaced and added regularly, and aphids were collected to form a stable population for reserve.

Luria Bertani (LB) medium: 5 g/L yeast powder (yeast extract), 10 g/L trypsin (tryptone), 10 g/L chlorination (NaCl); solid LB medium was prepared with 12 g/L agar powder added to the above formulation. Ampicillin and chloramphenicol were purchased from Shanghai Biotechnology Bioengineering Technology Service Co., Ltd. Shanghai, China with a final concentration of 100 μg/mL, 10 μg/mL, and pHT01-P43GFPmut3a was incubated in LB liquid medium at a final concentration of 100 μg/mL ampicillin and incubated overnight for plasmid DNA extraction using the Shanghai Production plasmid extraction kit. CAT, POD, PAL, and PPO kits were purchased from Suzhou King Biotechnology Co., Ltd., Suzhou, China

### 4.2. Natural Transformation of GFP-Harboring Vactor in the Bacterial Strains

The vector pHT01-P43GFPmut3a harboring gene-encoding green fluorescent protein was introduced to *Bacillus* cells following the two-step method provided by [48]. The freshly streaked *Bacillus* was transferred to the liquid LB medium and incubated at 37 °C with 160 rpm for 16 h. After incubation, 200 µL of the bacterial suspension was inoculated with 6 mL of the liquid GCHE medium. The cultures were incubated at 37 °C at 160 rpm for 4–5 h. When its OD_600_ reached approximately 1.4, the cell cultures were evenly divided into two portions, followed by centrifugation at 5000× *g* for 6 min at room temperature. Further, 2 mL of a transformation buffer and 50 ng of exogenous DNA were added to the precipitated bacterial suspension. After mixing well for 7 h at 37 °C at 90 rpm, 500 µL of chloramphenicol (8 μg/mL) was added and kept on a shaker for 3 h. A total of 300 µL of the supernatant was centrifugated at 10,000 rpm for 4 min and uniformly spread on an LB plate supplemented with 10 µg/mL of chloramphenicol, followed by incubation at 37 °C. No fluorescent colonies were observed the next day or the third day in a UV chamber. Successfully transformed single colonies were picked and cultured overnight in the LB liquid medium with a final concentration of 10 µg/mL chloramphenicol and stored in 40% glycerol (1:1) for future use.

### 4.3. Comparison of Strain Stability and Growth Curves

The *Bacillus* strain with a green fluorescence was cultured on LB agar supplemented with chloramphenicol. A single colony was obtained and streaked on the plate with the corresponding antibiotic, followed by overnight incubation at 37 °C at 160 rpm. In an LB broth of 0.1% (*v*/*v*), a culture was grown under the same conditions for 50 h. Before each transfer, the samples were serially diluted to a 10X gradient dilution and evenly spread on antibiotic-free LB plates for 16 h at 37 °C. The number of colonies that emitted the green fluorescence was then recorded under a UV chamber. The stability was checked by calculating the percentage of the total colonies. Single colonies of wild-type and fluorescent bacteria in the LB broth medium and corresponding antibiotics were cultured overnight at 37 °C at 160 rpm, the concentration was adjusted to OD_600_ of approximately 1.0 with fresh sterile LB broth, and the OD_600_ of the bacterial suspension was measured every 2 h; 8 to 21 h, every 3 h; 21 to 48 h, and 48 h. The growth curves of the different GFP-marked strains and the wild-type strains were constructed between the time of sampling at the horizontal axis and the OD_600_ values of the broth at the vertical axis.

### 4.4. External and Internal Colonization of AK-12 in Rapeseed Leaves

The fluorescent bacteria were cultured in an LB medium supplemented with chloramphenicol (10 µg/mL) at 37 °C and 160 rpm for 2 days. The bacterial concentration was adjusted to 1 × 10^9^ CFU/mL and 40 mL/basin was applied to 30 potted rapeseed plants. Subsequently, the colonization was tested for 30 days and sampling was carried out after 5 days. Three plants were randomly sampled from each treatment; the tissue homogenized with a sterilized mortar (0.1 g plus 900 µL of deionized water), diluted 10 times, and a corresponding dilution was evenly spread on the LB plates. In total, 100 µL from each concentration was spread on three LB agar plates (10 µg/mL chloramphenicol) and three dilutions were selected for the experiment. The number of fluorescent colonies was counted the next day under a fluorescent inverted microscope. In order to check the internal colonization of strains, sampled leaves were surface sterilized with an alcohol gradient and 2.5% sodium hypochlorite for 2 min, followed by rinsing with sterile water. After the surface sterilization, the aforementioned process was carried out. A sterility check was performed by plating 100 µL of water from the last wash. The plates were incubated at 37 ℃ overnight and colonies were counted under a UV chamber.

### 4.5. AK-12 Colonization Ability to Establish Green Barrier against Aphids

#### 4.5.1. Effect of AK-12 on Aphid Transmission

**Figure 11:**
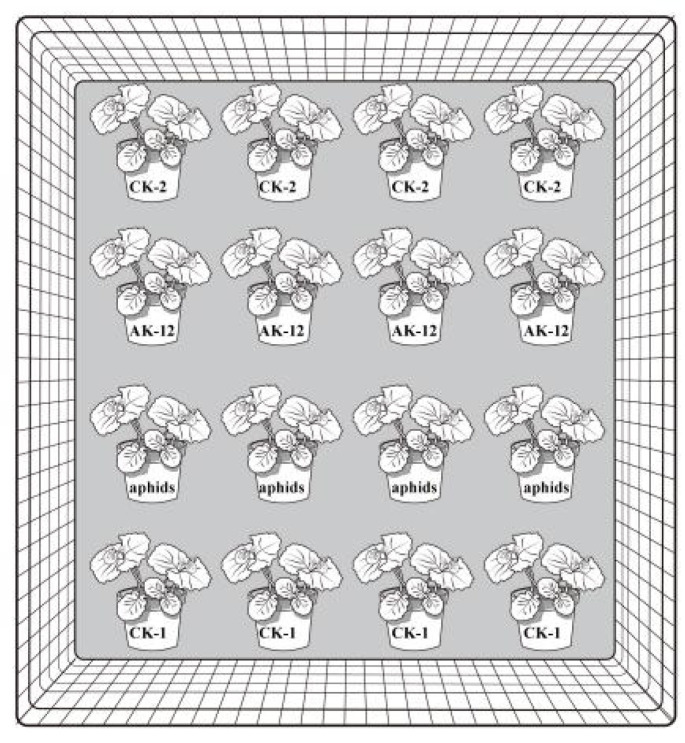
Graphical illustration of aphid and AK-12 treatment applied to rapeseed plants for transmission study.

In order to explore the effect of the bacterial suspension (AK-12-S) treatment on aphids, the rapeseed seedlings were arranged in four rows and treated with AK-12S (1 × 10^9^ CFU/mL, 40 mL/basin). Further, 100 aphids with consistent vitality and body size were inoculated to each pot in the third row (Figure 11). The number of aphids in the rapeseed seedlings was recorded regularly to observe the transmission pattern.

#### 4.5.2. Effect of Strains on Aphid Feeding

**Figure 12:**
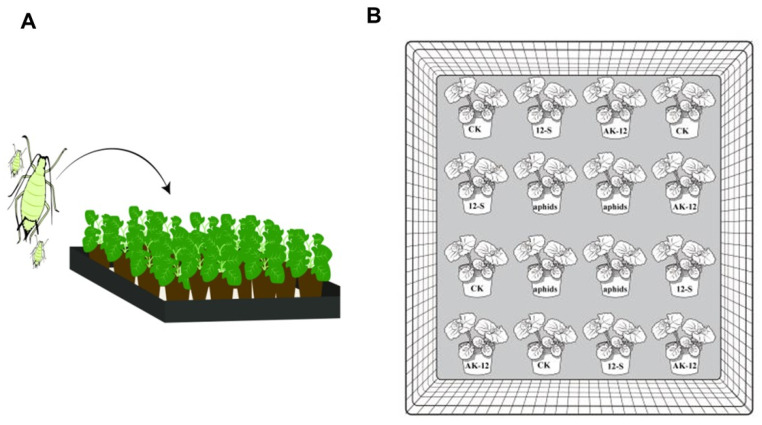
Aphid infestation and treatment combinations to rapeseed. (**A**) Illustration showing the aphid application to rapeseed seedlings arranged in rows. (**B**) The arrangement of rapeseed pots and treatment combinations.

After the seed germination, 5 seedlings were transplanted into one pot. The pots were placed in a greenhouse for 20 days and, then, the potted rapeseed seedlings with the same growth rate were selected for a further experiment. The pots were treated and arranged into 4 combinations and placed in the greenhouse to ensure the same spacing (10 cm), with 4 pots being used for each treatment (Figure 12). A wild-type culture solution was sprayed on line 2, following the method mentioned in Section 4.5.1, line 3 was inoculated with aphids, and the rest was sprayed with equal amounts of sterile water, and two peripheral rows served as the controls. In total, 100 aphids with a consistent body size were inoculated in each basin (Figure 11). The number and movement of aphids in the rapeseed seedlings were recorded every 10 days.

### 4.6. Growth Promotion in Rapeseed Treated with AK-12

After 24 h of culture incubation, 10 µL of the bacterial solution was inoculated in nitrogen-free solid medium, PKO inorganic media, and dissolved potassium screening medium to detect solid N, dissolved P, and K activity [49]. The quantitative determination of the IAA concentration was performed following Glickman. et al., 1995 [50]. Different strains were cultured for 24 h at 37 °C and 160 r/min. The culture was centrifuged at 10,000 r/min for 10 min, followed by the mixing of 4 mL of a supernatant with an equal volume of Salksowski colorimetric solution. It was kept for 30 min and OD_530_ was recorded. The rapeseed seedlings at the two-leaf stage were treated with AK-12 (1 × 10^9^ CFU/mL, 40 mL/pot) through root irrigation, foliar spraying, and root irrigation combined with foliar spraying. Each treatment was performed in triplicate and sterile water was applied as a control. After 30 days of treatment, the physiological traits were measured.

### 4.7. Study of Induced Resistance in Rapeseed Due to AK-12

To determine the effect of AK-12 on rapeseed resistance, the plants were treated with 1 × 10^9^ CFU/mL, 40 mL per each pot, and aphids were infested later, as mentioned in Section 4.5.1. After 30 days, the leaves were sampled from treated and control plants of the same age to check the activity of defense enzymes, including peroxidase (POD), phenylalanine ammonia lyase (PAL), polyphenol oxidase (PPO), and catalase (CAT), at 6 h, 12 h, 24 h, 36 h, and 48 h. To further study the expression level of the resistance-related genes, the rapeseed seedlings were treated after 30 days with bacterial broth (1 × 10^9^ CFU/mL) for 0 h, 6 h, 12 h, 24 h, 48 h, and 72 h. Three replicates were used for each treatment, each with 5 rapeseed seedlings. The genes involved in defense activation, such as NPR1 (nonexpressor of pathogenesis-related gene1), plant defensin gene PDF 1.2, and chitinase encoding Chit gene, and genes encoding defense-related enzymes, such as phenylalanine ammonia lyase PAL, peroxidase POD, and polyphenol oxidase PPO, were investigated.

The quantitative PCR primers were designed according to the reference gene tubulin and target genes (Table 2). RNA was extracted using the *TransZol*^TM^Up RNA kit, according to the operating instructions of Dongyang Textile, Biotechnology Co., Ltd. (Shanghai, China). A Rever Tra Ace qPCR RT Kit was used to reverse transcribe the RNA into cDNA and the concentration was quantified to 100 ng/mL by using a Nanodrop. Relative fluorescence quantitative PCR reactions were performed using the SYBR ^®^ Green I fluorescent dye method, following the instructions of the Toyo Spinning SYBR ^®^ Green Realtime PCR Master Mix.

### 4.8. Data Statistics and Analysis

SPSS23.0 and GraphPad Prism 9.4.0 were used for the data analysis and the plots were designed using Adobe Illustrator 2020 (24.0.2).

## Figures and Tables

**Table 1:** Different growth parameters of rapeseed after AK-12 treatment through root irrigation, foliar spray, and both.

	Stem Length (cm)	Stem Thick (mm)	Fresh Weight (g)	Dry Weight (g)
Foliar spray	27.92 ± 1.98 ^ab^	3.62 ± 0.29 ^ab^	4.06 ± 0.51 ^ab^	0.46 ± 0.12 ^ab^
Irrigation	30.7 ± 2.68 ^a^	4.06 ± 0.31 ^a^	5.17 ± 0.63 ^a^	0.59 ± 0.06 ^a^
Foliar spraying and irrigation	29.82 ± 1.81 ^a^	4.08 ± 0.32 ^a^	5.5 ± 1.49 ^a^	0.65 ± 0.17 ^a^
CK	24.6 ± 0.76 ^b^	3.08 ± 0.28 ^b^	2.69 ± 0.20 ^b^	0.28 ± 0.05 ^b^

Note: Different letters in column indicate statistical differences (*p* < 0.05).

**Table 2:** The primers used in this study.

Primers	Primer-F Sequence (5′-3′)	Primers-R Sequence (5′-3′)
Tubulin	GAGCGACCCACATACACCAATC	AACCTCAACGAAGCAGTCAACG
NPR1	ACGCTTCTTTCCACGATGTTCAG	GCTTCTTCAGTTGACGCTCTTCC
PDF1.2	GGGACCATGCTCAAGAGACAG	AACAACGGCGGCGGAATC
chit	TCGGCAGTATCATCTCAAGTTCC	TTTACGGGCAGTGGTATCGC
POD	ACACACATTTGGAAGAGCAAGATG	CGTCTACGGTTGGATCAGGATTAC
PPO	TGGGTTTAGGAGGGCTGTATGG	TGAGATCAGGAGGTGGTATAGGAG
PAL	CGGTTTGCCCTCTAATCTCACTG	GACATCTTGGTTGTGTTGTTCAGC

Note: In the names of the primers, F and R stand for forward (F) and reverse (R) primers, respectively. All the sequences were generated in the present study.

## Data Availability

All the data are available in the manuscript text.
